# Magnolol Suppresses TGF-β-Induced Epithelial-to-Mesenchymal Transition in Human Colorectal Cancer Cells

**DOI:** 10.3389/fonc.2019.00752

**Published:** 2019-10-01

**Authors:** Sungwoo Chei, Hyun-Ji Oh, Ji-Hyeon Song, Young-Jin Seo, Kippeum Lee, Boo-Yong Lee

**Affiliations:** Department of Food Science and Biotechnology, College of Biomedical Sciences, CHA University, Seongnam, South Korea

**Keywords:** tumor metastasis, colorectal cancer, magnolol, epithelial-to-mesenchymal transition, TGF-β

## Abstract

Tumor metastasis is the end state of a multistep process that includes dissemination of tumor cells to distant organs and requires tumor cells to adapt to different tissue microenvironments. During metastasis, tumor cells undergo a morphological change known as transdifferentiation or the epithelial-to-mesenchymal transition (EMT). In normal embryonic development, the EMT occurs in the context of morphogenesis in a variety of tissues. Over the course of this process, epithelial cells lose their cell–cell adhesion and polarity properties. In this study, we investigated whether magnolol could suppress the EMT in human colorectal cancer cells. To this end, we examined the epithelial markers E-cadherin, ZO-1, and claudin and the mesenchymal markers N-cadherin, TWIST1, Slug, and Snail. Magnolol effectively inhibited EMT in human colon cancer cell lines by upregulating epithelial markers and downregulating mesenchymal markers. The EMT is induced by the TGF-β signaling pathway. To determine whether magnolol disrupts TGF-β signaling, we examined several mediators of this pathway, and found that magnolol decreased the levels of phosphorylated (i.e., active) ERK, GSK3β, and Smad. We conclude that magnolol blocks migration in HCT116 cells by suppressing TGF-β signaling.

## Introduction

Although colon carcinoma is among the best-understood malignancies, it remains a common cause of cancer-related death. Due to advances in surgical techniques, mortality from colon cancer has declined significantly (1). However, surgical resection is exclusively used to treat localized tumors, and is therefore limited to the early stage of colon cancer progression (2). Currently, no effective therapy is available for metastatic colon cancer; consequently, despite improvements in systemic therapy and radiotherapy over the past few years, the disease continues to have a high mortality rate. Therefore, it is necessary to expand efforts aimed at blocking metastasis. In particular, it is important to identify natural substances, which are less likely to have deleterious side effects, that can prevent cancer cells from spreading.

Many bioactive compounds from plants have been considered as potential cancer therapeutics. Magnolol is a common bioactive component isolated from the bark of the Houpu magnolia (*Magnolia officinalis*) (3). Magnolia bark is a traditional ingredient in Chinese and Japanese folklore medicine and is still used today (4). Magnolol has a wide range of biological activities, including anti-oxidative (5), anti-microbial (6), anti-atherosclerotic (7), and anti-inflammatory effects (8), but it remains unknown whether it has anti-tumor activity. In this study, we obtained evidence that magnolol has potent activity against colon cancer.

Apoptosis is a major form of cell death, used by multicellular organisms to eliminate cells and control cell proliferation (9). Apoptosis is often inhibited in cancer, resulting in elevated proliferation and a reduction in cell death (10). A deeper understanding of apoptosis has provided the basis for targeted therapies that can induce death in cancer cells. Multiple studies have shown that defects in apoptotic pathways play significant roles in carcinogenesis, and that many therapeutic strategies focused on apoptosis could be used to treat several types of cancer (11, 12). However, apoptosis-inducing cancer therapy causes several side effects (13), including damage to normal cells and reduced production of blood cells and platelets in the bone marrow (14). Accordingly, we examined the anti-tumor effect of magnolol, focusing on inhibiting metastasis rather than promoting apoptosis.

Tumor metastasis involves several steps, over the course of which tumor cells spread from their primary site and develop into secondary tumors at remote sites (15). The epithelial-to-mesenchymal transition (EMT) is an important physiological and morphological process in cancer cell metastasis (16). During the EMT, epithelial cells forfeit their polarized phenotype and downregulate cell–cell adhesion molecules, and eventually acquire mesenchymal traits that help the cells to scatter (17). The EMT is a complex multistep process involving altered expression of many epithelial and mesenchymal genes (18). Epithelial cadherin (E-cadherin), Zona occludens-1 (ZO-1), and claudin are the best-characterized markers expressed in epithelial cells (19–21), whereas neural cadherin (N-cadherin), Twist-related protein 1 (TWIST1), Slug, and Snail are expressed in mesenchymal cells (22, 23). The loss of epithelial markers and gain of mesenchymal markers are correlated with cancer stage (24). Because changes are closely linked to disease progression, it is crucial to monitor the expression of EMT markers.

The EMT is regulated by several signaling pathways, in particular transforming growth factor β (TGF-β). TGF-β downregulates E-cadherin, ZO-1, and claudin and upregulates N-cadherin, TWIST1, Slug, and Snail (25). TGF-β is also the major inducer of the EMT in lung (26), pancreatic (27), esophageal (28), breast (29), and colon cancers (30). Signaling molecules such as extracellular signal-regulated kinases (ERKs), glycogen synthase kinase 3 beta (GSK3β), and Smad are also differentially regulated during the TGF-β-induced EMT (31–33). The expression of these molecules allows us to predict the EMT suppression through the blocking of TGF-β signaling pathway. TGF-β induces the EMT through a cell cycle–dependent mechanism in which the transition occurs in G1/S phase (27). Thus, cell growth arrest at G1/S phase may be a prerequisite for undergoing the EMT and blocking the TGF-β-induced growth arrest at G1/S by treating cancer cells with magnolol could prevent the EMT.

In this study, we investigated the potential of magnolol to suppress EMT as well as the mechanism underlying the effect of cell cycle–dependent TGF-β signaling. In addition, to address concerns about the side effects of apoptosis-inducing therapy, we confirmed that magnolol does not affect programmed cell death in human colon cancer cells. Together, our results provide strong evidence that magnolol is a bioactive compound with potent anti-cancer effects, specifically the ability to suppress tumor metastasis.

## Materials and Methods

### Materials and Chemicals

Human colorectal adenocarcinoma cell lines HCT116 and SW480 were purchased from American Type Culture Collection (ATCC; Manassas, VA, USA). Antibodies against PARP-1 (sc-8007), PCNA (sc-56), α-tubulin (sc-5286), ZO-1 (sc-33725), p-GSK3β (sc-135653), and Twist (sc-15393) were purchased from Santa Cruz Biotechnology (Dallas, TX, USA). Antibodies against claudin (#132552), p-Akt (#9271), Slug (#9585), Snail (#3879), N-cadherin (#13116), E-cadherin (#3196), and p-Smad (#9511) were purchased from Cell Signaling Technology (Beverly, MA, USA). Recombinant human TGF beta 1 protein (TGF-β) was purchased from Abcam (Cambridge, UK).

### Cell Culture and Treatment

Cells were maintained in Dulbecco's modified Eagle's medium (DMEM; Sigma-Aldrich, St. Louis, MO, USA) supplemented with 10% fetal bovine serum, FBS (Sigma-Aldrich), and 1% penicillin–streptomycin at 37°C in the presence of 5% CO_2_. Magnolol was dissolved in DMSO (Sigma-Aldrich) and filter-sterilized using a 0.22 μm filter (Millipore, Billerica, MA, USA). DMSO solution without magnolol was used as vehicle. When the cell seeded, cells were washed and cultured in serum-free medium containing magnolol.

### Magnolol Compositional Analysis

Magnolol, Honokiol, and Magnolia bark extract was purchased from Rongsheng (Shaanxi, China). The composition of the extract was analyzed by high-performance liquid chromatography (HPLC). Chromatic separation was conducted at 30°C on a Luna® 5 μ phenyl-hexyl column (250 × 4.6 mm; particle size, 5 μm), after which samples were eluted using a mobile phase composed of 75% acetonitrile and 25% 0.1% TFA water, which was applied for 30 min. Analytes were detected by UV at 290 nm. The analysis was replicated three times.

### Western Blotting

Cells were washed with PBS, and cell lysates were isolated in RIPA buffer (50 mM Tris-HCl [pH 7.4], 150 mM NaCl, 1 mM EDTA, 1% Triton X-100, 1% sodium deoxycholate, and 0.1% SDS) supplemented with protease inhibitors (1 mM PMSF, 5 μg/mL aprotinin, and 5 μg/mL leupeptin) and phosphatase inhibitors (1 mM Na3VO4 and 1 mM NaF) and centrifuged at 12,000 rpm for 5 min at 4°C. Protein concentrations were determined by BCA protein assay (Pierce, Rockford, IL, USA) using BSA as the standard. Proteins (20 μg/lane) were separated by SDS-PAGE and transferred to Immun-Blot PVDF membranes (Bio-Rad, Hercules, CA, USA). The membranes were incubated at 4°C overnight with specific primary antiserum in Tris-buffered saline (TBS) containing 0.05% Tween-20 (TBS-T) and 5% non-fat dry milk. After three washes with TBS-T, membranes were incubated for 1 h with peroxidase-conjugated anti-rabbit or anti-mouse IgG at room temperature. Signals were visualized using EZ-Western Lumi Femto (DoGenBio, Seoul, Korea) and quantified on an LAS-4000 (GE Healthcare Life Sciences, Marlborough, MA, USA). Densitometry of protein bands was performed using the ImageJ software (34) (Bethesda, MD, USA).

### RNA Isolation and Quantitative Real-Time PCR (qRT-PCR)

Total RNA was extracted from cells using Trizol reagent (Invitrogen, Carlsbad, CA, USA). Extracted RNA was reverse transcribed to cDNA using the Maxime RT PreMix kit (Intron, Seongnam, Korea). The sequence of the oligonucleotide primer was as follows: E-cadherin 5′-TGCCCAGAAAATGAAAAAGG-3′ (sense) and 5′-GTGTATGTGGCAATGCGTTC-3′ (antisense); N-cadherin 5′-CAAGCCCTTTGAGCCAAGAAG-3′ (sense) and 5′-GCTGTAGACGTGAGGTAGGTAG-3′ (antisense); Slug 5′-TGTGACAAGGAATATGTGAGCC-3′ (sense) and 5′-TGAGCCCTCAGATTTGACCTG-3′ (antisense); Snail 5′-ACTGCAACAAGGAATACCTCAG-3′ (sense) and 5′-GCACTGGTACTTCTTGACATCTG-3′ (antisense); TWIST1 5′-GTCCGCAGTCTTACGAGGAG-3′ (sense) and 5′-CCAGCTTGAGGGTCTGAATC-3′ (antisense); Claudin-1 5′-CCCGGTCAATGCCAGATATG-3′ (sense) and 5′-CACCTCCCAGAAGGCAGAGA-3′ (antisense); ZO-1 5′-TGCTGAGTCCTTTGGTGATG-3′ (sense) and 5′-AATTTGGATCTCCGGGAAGAC-3′ (antisense); GAPDH 5′-CAGAACTACATCCCTGCATC-3′ (sense) and 5′-CCACCTTCCTGATGTCATCA-3′ (antisense). The level of GAPDH mRNA was used as the control. qPCR was performed using Mx3005P qPCR System (Agilent Technologies, CA, USA).

### Conventional RT-PCR

Total RNA was analyzed using T100™ Thermal Cycler (Bio-Rad, Hercules, CA, USA). The sequence of the oligonucleotide primer was as follows: TGF-β1 5′-ATGACATGAACCGACCCTTC-3′ (sense) and 5′-ACTTCCAACCCAGGTCCTTC-3′ (antisense); TGF-β R1 5′-ACCTTCTGATCCATCCGTT-3′ (sense) and 5′-CGCAAAGCTGTCAGCCTAG-3′ (antisense); GAPDH 5′-CAGAACTACATCCCTGCATC-3′ (sense) and 5′-CCACCTTCCTGATGTCATCA-3′ (antisense). The level of GAPDH mRNA was used as the control. PCR products were run on 1.5% agarose gels, stained with ethidium bromide and photographed.

### Cell Migration Assay

HCT116 cells were seeded in 6-well plates and cultured to confluence. After 24 h, scratches were made using 1 mL pipette tips, and cellular debris was removed by gentle washes with culture medium. Cells were allowed to migrate for an additional 72 h. Photographs were taken at 48 h and 72 h after cell seeding using an Olympus CKX53 inverted microscope (Olympus, Tokyo, Japan). The perimeter of the scratched area was automatically selected, and the area quantified using the “Measure” function of ImageJ, and the data was normalized to the average of 0 h values.

### Single-Cell Invasion Assay

Cell invasion assays were performed using Transwell chambers (24-well; 8 μm pore size; Corning, New York, NY, USA). About 5 × 10^5^ cells in DMEM medium containing 0.5% serum were added to the upper compartment of the insert and allowed to migrate toward the underside of the insert filter at 37°C for 48 h. Cells that did not migrate through the pores were gently removed with a cotton swab. Cells on the lower side of the insert filter were fixed with 5% glutaraldehyde and stained with 1% crystal violet in 2% ethanol for 10 min. Cells on the underside of the filter were counted in randomly selected microscopic fields.

### Cell-Cycle Analysis

Cells were collected by trypsinization, washed twice with PBS, and centrifuged at 1,000 rpm for 5 min. After washing with PBS, the cells were fixed in 70% ethanol for 24 h at 20°C. The cells were then centrifuged, washed again with PBS, and suspended in 500 μL of PBS. The cells were incubated with RNase A from bovine pancreas (Sigma-Aldrich); the working solution was made by diluting the 10 mg/mL stock solution 1:2000 in PBS. The cells were then stained for 3 h at 4°C with propidium iodide (PI; Thermo Fisher, Waltham, MA, USA); the working solution was made by diluting the 1 mg/mL stock solution 1:2000 in PBS. Cell-cycle distribution was determined by flow cytometry (FACS Calibur; BD Biosciences, Franklin Lakes, NJ, USA), and the data were analyzed with FlowJo (Ashland, OR, USA).

### Apoptosis Assay

Cells (1 × 10^6^) were collected with trypsin-EDTA, washed twice with PBS, and then fixed in 70% ethanol for 24 h at 20°C. The cells were then centrifuged and washed with 200 μL of 1× binding buffer from the ApoAlert Annexin V–FITC Apoptosis kit (cat #630109; Clontech, Mountain View, CA, USA). The cells were incubated for 5–15 min in the dark with 5 μL of Annexin V solution from the kit. After staining, the cells were washed with PBS. The cell-cycle distribution was determined by flow cytometry on a Guava easyCyte (Luminex, Austin, TX, USA) and analyzed using the FlowJo software.

### MTT Assay

Cells (5 × 10^3^ cells/well in a 96-well plate) were incubated overnight with DMEM with 10% FBS and 1% penicillin–streptomycin. The cells were then treated with magnolol (0, 1.25, 2.5, 5, 10, or 20 μM) and cultured for an additional 24 h. Then, MTT reagent was added to the 96-well plate and incubated for 3 h at 37°C. Supernatant was gently removed, and 100 μL of DMSO was added to extract intracellular formazan. MTT-formazan product was measured on a PowerWaveHT ELISA reader (BioTek, Winooski, VT, USA) at 570 nm.

### Statistical Analysis

Data are represented as means ± standard deviation (SD) of triplicate experiments. Comparisons were evaluated by one-way analysis of variance (ANOVA) followed by Tukey's multiple range tests; statistical analyses were performed in SPSS (IBM, Armonk, NY, USA). A value of *p* < 0.05 was considered to indicate a statistically significant difference.

## Result

### Magnolol Does Not Affect Apoptotic Cell Death, but Suppresses the EMT in HCT116 Cells

**Figure 1:**
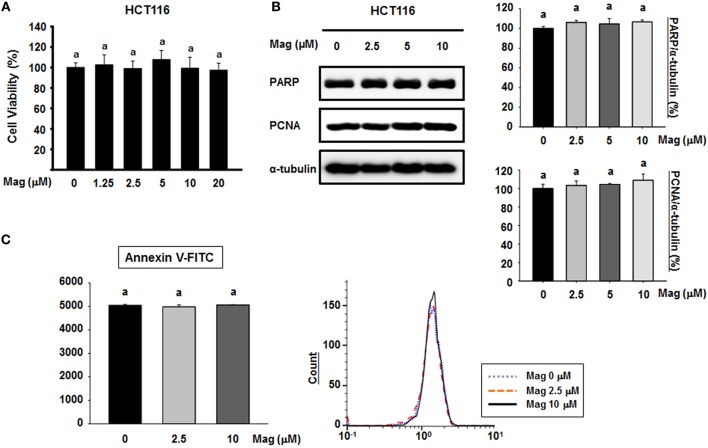
Cytotoxicity of magnolol and its effect on apoptosis in HCT116 cells. **(A)** HCT116 cells were treated for 24 h with 0, 1.25, 2.5, 5, 10, or 20 μM magnolol in medium containing 1% serum. Cell viability was assessed after 24 h by MTT assay. Experiments were repeated five times independently to confirm reproducibility; standard deviation of the mean is indicated by error bars (*n* = 5). **(B)** HCT116 cells were treated with 0, 2.5, 5, or 10 μM magnolol for 24 h. Western blots were performed for apoptosis-associated proteins PARP and PCNA. α-tubulin was used as an internal control. **(C)** HCT116 cells were treated with 0, 2.5, or 10 μM magnolol for 24 h. Cells were examined by flow cytometry. In **(A,C)**, values labeled with the letter a do not differ significantly (i.e., *p* > 0.05).

To determine the cytotoxic effect of magnolol, we treated HCT116 cells with various concentrations of magnolol (0–20 μM) for 24 h. Cell viability was not significantly affected by any concentration of magnolol (Figure 1A), so we selected concentrations of 0, 2.5, 5, and 10 μM for subsequent experiments. To determine whether magnolol induces apoptosis in HCT116 cells, we exposed the cells to magnolol (0, 2.5, 5, or 10 μM) for 24 h, and then performed western blot for poly (ADP-ribose) polymerase (PARP) and proliferating cell nuclear antigen (PCNA), both of which are associated with apoptosis. Regardless of magnolol concentration, cleaved PARP fragment was not detected and expression of PAPR and PCNA remained constant (Figure 1B). In addition, we analyzed apoptosis by flow cytometry; in these experiments, detection was based on binding of Annexin V–FITC to phosphatidylserine (PS) in the cell membrane. All three concentrations of magnolol yielded similar flow cytometry histograms (Figure 1C). Thus, magnolol did not affect apoptosis in HCT116 cells.

**Figure 2:**
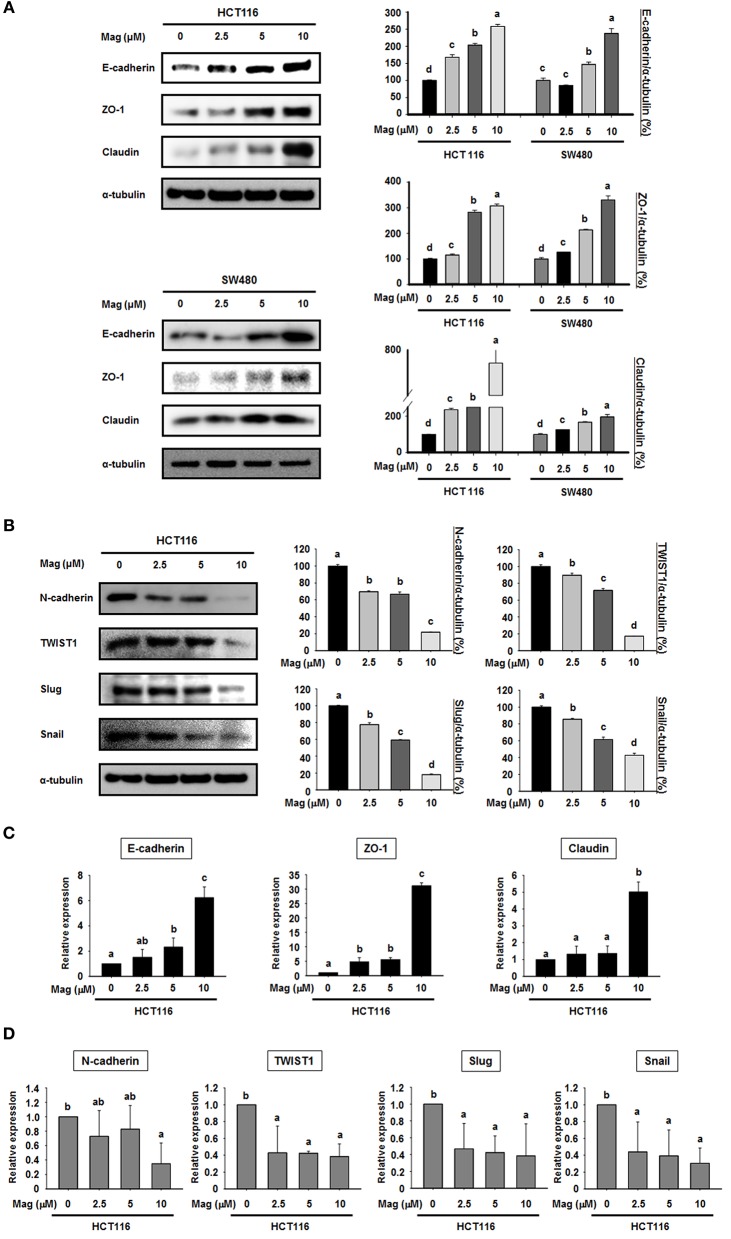
Magnolol regulates the expression of EMT marker genes in human colon cancer cells. **(A)** HCT116 and SW480 cells were treated with 0, 2.5, 5, or 10 μM magnolol for 24 h, and western blots were performed for E-cadherin, ZO-1, Claudin, and α-tubulin (used as an internal control). **(B)** HCT116 cells were treated with 0, 2.5, 5, or 10 μM magnolol for 24 h, and western blots were conducted for N-cadherin, TWIST1, Slug, Snail, and α-tubulin. **(C)** mRNA expression of E-cadherin, ZO-a, and Claudin in HCT116 cells treated with magnolol (0, 2.5, 5, or 10 μM) for 24 h. **(D)** mRNA expression of N-cadherin, TWIST1, Slug, and Snail in HCT116 cells treated with magnolol (0, 2.5, 5, or 10 μM) for 24 h. In **(C,D)**, GAPDH served as a control. All data values labeled with different letters are significantly different (*p* < 0.05).

Given the lack of an effect on apoptosis, we next explored the possibility that magnolol influences the EMT in colon cancer cells. To this end, we performed western blots for EMT biomarkers in the primary colon cancer cell lines HCT116 and SW480. After treatment with magnolol (0, 2.5, 5, or 10 μM) for 24 h, the expression of epithelial markers (E-cadherin, ZO-1, and claudin) was increased in a concentration-dependent manner in both cell lines (Figure 2A), whereas the expression of mesenchymal markers (N-cadherin, TWIST1, Slug, and Snail) was decreased in a concentration-dependent manner in HCT116 (Figure 2B). We used qRT-PCR to confirm the expression levels of EMT marker genes (Figures 2C,D), and the result was same as the western blot result. Thus, magnolol inhibited the EMT in human colon cancer cells.

### Magnolol Inhibits Cell Migration and Blocking the TGF-β Signaling Pathway in HCT116 Cells

**Figure 3:**
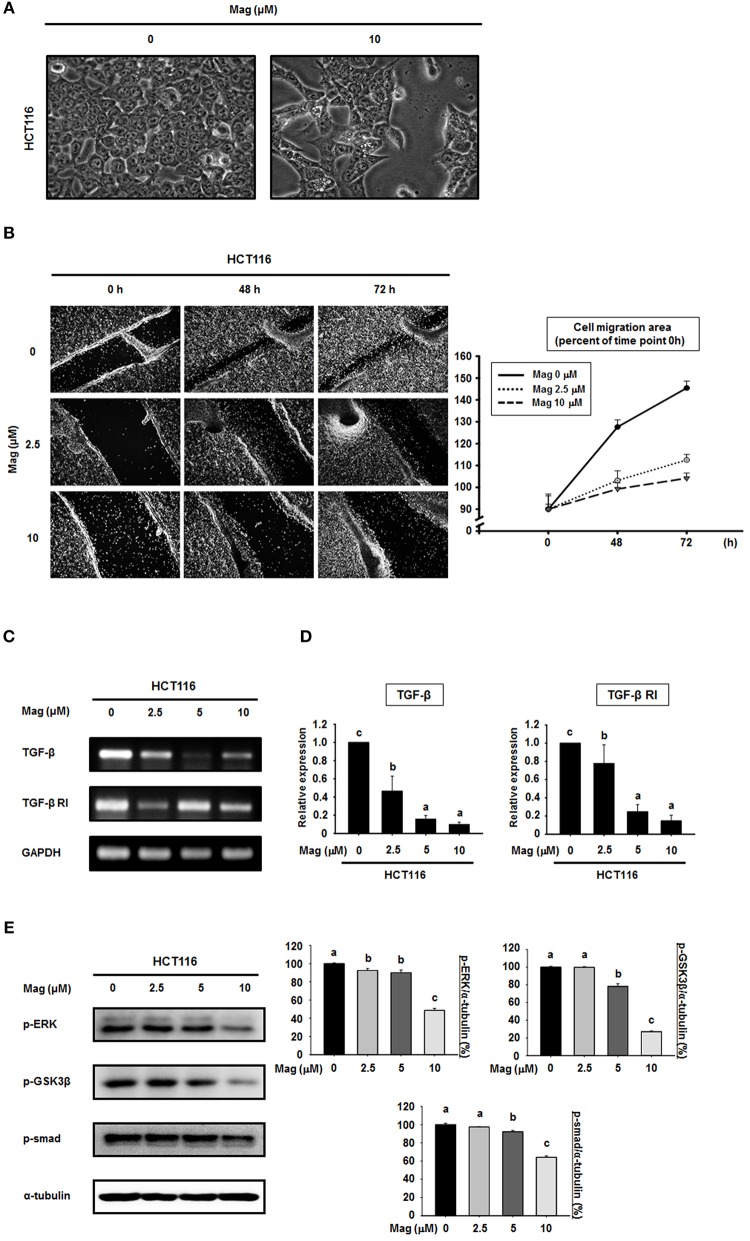
Cell migration is suppressed and the TGF-β signaling pathway is blocked in HCT116 cells by magnolol. **(A)** The morphological changes in HCT116 cells treated with or without magnolol (10 μM). Images were taken at 48 h after treatment using a microscope. **(B)** Confluent monolayers of HCT116 cells were scratched using a pipette tip prior to treatment with 0, 2.5, or 10 μM magnolol. The area of migration was measured at 0, 48, and 72 h. Photographs were taken at 48 and 72 h after cell seeding using a microscope. The area quantified using ImageJ, and the data was normalized to the average of 0 h values. (**C**) RT-PCR analysis of TGF-β and TGF-β RI expression in HCT116 cells treated with (0, 2.5, 5, or 10 μM) for 24 h. GAPDH was used as control. (**D**) Quantitative RT-PCR analysis of TGF-β and TGF-β RI expression in HCT116 cells treated with (0, 2.5, 5, or 10 μM) for 24 h. The graphs show the mRNA expression of TGF-β and TGF-β RI relative to GAPDH. **(E)** HCT116 cells were treated with the indicated concentrations of magnolol for 24 h, and western blots were performed for p-ERK, p-GSK3β, p-Smad, and α-tubulin. Data values labeled with different letters are significantly different (*p* < 0.05).

Given that magnolol suppressed the EMT in HCT116 cells, we first observed whether magnolol inhibits alteration of HCT116 cells to EMT cell morphology by light microscopy. HCT116 cells were incubated for 48 h, and the cells changed their shape from a typical epithelial to fibroblast-like appearance. However, after treatment with magnolol (10 μM) markedly enhanced cell-to-cell contact, and the cells formed a more rounded shape (Figure 3A). We next investigated the influence of magnolol on cell migration. For this purpose, the cells were cultured until confluence, scratched with a pipette tip, treated with magnolol (0, 2.5, or 10 μM), and allowed to migrate for 72 h. The cell migration assay revealed that magnolol inhibited cell migration, as determined by measuring the migration area covered by cells from 0 to 72 h in a dose-dependent manner (Figure 3B).

Because the EMT can be controlled by the TGF-β signaling pathway, we sought to determine whether magnolol treatment affects TGF-β signaling. The mRNA expression of TGF-β and TGF-β receptor I (TGF-β RI) was examined using RT-PCR and qRT-PCR (Figures 3C,D). RT-PCR analysis showed the down-regulation of TGF-β and TGF-β RI gene expressions compared to control after treatment with magnolol (Figure 3C). The qRT-PCR analysis showed the similar results (Figure 3D). Moreover, we treated cells with magnolol (0, 2.5, 5 or 10 μM) and performed western blots for p-ERK, p-GSK3β, and p-Smad, all of which are downstream proteins of the TGF-β signaling pathway. As shown in Figure 3E, magnolol consistently decreased the expression of these downstream target proteins. In particular, at 10 μM magnolol, expression of the entire TGF-β signaling pathway was significantly reduced.

### Magnolol Regulates TGF-β-Induced EMT Signaling in HCT116 Cells

**Figure 4:**
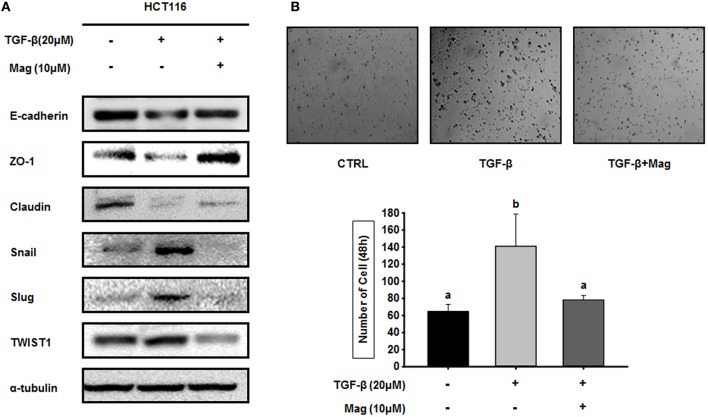
Magnolol blocks TGF-β-induced EMT signaling. **(A)** HCT116 cells were treated with TGF-β (20 μM) in the presence or absence of magnolol (10 μM), and western blots were performed for the indicated EMT-related proteins. α-tubulin was used as an internal control. **(B)** Single-cell invasion assay of HCT116 cells treated with TGF-β (20 μM) in the presence or absence of magnolol (10 μM). Cells were allowed to migrate for 48 h after treatment. Microscopic fields were randomly selected (*n* = 3). Values labeled with different letters are significantly different (*p* < 0.05), and the standard deviation of the mean is indicated as error bars (*n* = 3).

TGF-β induces the EMT by upregulating epithelial marker proteins and downregulating mesenchymal marker proteins. Hence, we investigated the role of magnolol in the TGF-β-induced EMT. When we treated HCT116 cells with TGF-β (20 μM) in the absence of magnolol, the expression of epithelial marker proteins (E-cadherin, ZO-1 and claudin) was reduced, whereas expression of mesenchymal marker proteins (Snail, Slug, and TWIST1) was elevated. However, when we treated cells with TGF-β (20 μM) in the presence of magnolol, we obtained the opposite results: epithelial marker proteins were upregulated relative to the control, and mesenchymal marker proteins were downregulated (Figure 4A). Because magnolol inhibited the TGF-β-induced EMT in HCT116 cells, we investigated the role of magnolol in TGF-β-induced cancer cell invasion. TGF-β stimulated invasion by HCT116 cells, whereas magnolol decreased the number of invading cells to control levels (Figure 4B). Together, these data demonstrated that magnolol suppresses the TGF-β-induced EMT and TGF-β-induced cell invasion.

### Magnolol Inhibits the TGF-β-Induced EMT by Altering the Cell Cycle Distribution in HCT116 Cells

**Figure 5:**
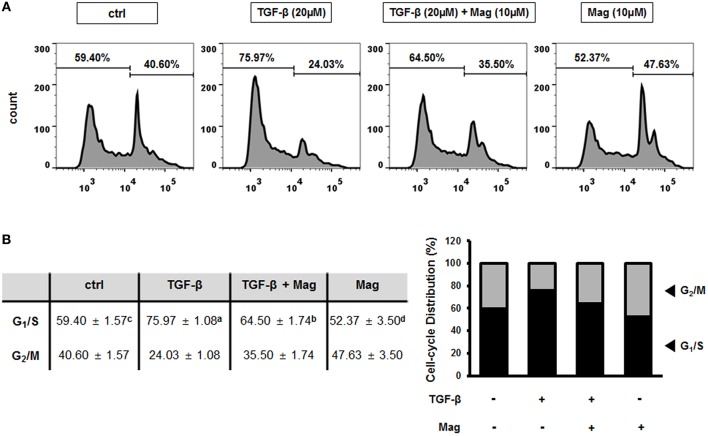
Magnolol prevents the TGF-β-induced EMT by inhibiting cell-cycle arrest at G1/S phase. **(A)** Cell cycle measurements using flow cytometry after exposure to TGF-β (20 μM) for 24 h, TGF-β (20 μM) for 3 h and magnolol (10 μM) for 18 h, and magnolol (10 μM) for 24 h. Representative cell cycle profiles are shown (*n* = 3). **(B)** Cell-cycle distributions, shown in table (b, left) and column graph format (b, right). Data values labeled with different letters are significantly different (*p* < 0.05).

We previously showed that the TGF-β-induced EMT is related to the cell cycle, in particular G1/S phase (27). To determine whether magnolol affects cell cycle–dependent TGF-β signaling in HCT116 cells, we assessed the proportion of cells in various phases of the cell cycle by flow cytometry. When HCT116 cells were treated with TGF-β (20 μM), the number of cells at G1/S phase increased relative to the control, but treatment of TGF-β (20 μM) with magnolol (10 μM) had the opposite effect. In addition, the number of cells in G1/S phase decreased further when magnolol was treated alone (Figure 5A). In quantitative terms, the number of cells in G1/S phase increased from 59.40% (ctrl) to 75.97% when TGF-β was applied, indicating induction of the EMT. However, when the cells were treated with TGF-β after magnolol exposure, the number of cells in G1/S phase dropped to 64.50%. The number of cells in G1/S phase further dropped to 52.37% when the cells were treated with magnolol alone (Figure 5B left). Graphical analysis yielded the same result (Figure 5B right). Thus, TGF-β induces EMT by arresting the cell cycle at G1/S, and magnolol suppresses the EMT by inhibiting this arrest.

## Discussion

Anti-cancer therapies have been studied for decades, and the most common mechanism of action of these drugs is induction of apoptosis (35). Several drugs activate caspases, thereby inducing apoptosis in cancer cells (36). However, according to recent studies, chemical-induced apoptosis can cause brutal side effects, including damage to normal cells (13). Despite efforts to minimize damage to normal cells, side effects of apoptosis-inducing treatments continue to be reported. Hence, we focused on curbing the spread of cancer cells, the other common mechanism of anti-cancer therapies. Magnolol, a natural bioactive compound, is known to have several biological activities, but the effect on metastasis of cancer cell is not well-known. We suggested that novel target for magnolol is TGF-β, which is inducer of metastasis in colon cancer cell. Magnolol represents a cancer treatment that does not affect apoptosis, but instead inhibits cancer cell metastasis.

Several studies have evaluated apoptosis in cancer cells using apoptotic markers (37). PARP and PCNA play central roles in cell death. Hyperactivation of PARP results in specific programmed cell death and PARP is cleaved by enzymes such as caspases of cathepsins during apoptosis (38), whereas a low level of functional PCNA drives cells into apoptosis (39). Our data revealed that magnolol does not affect apoptosis, as reflected by the constant levels of PARP and PCNA when HCT116 cells are treated with magnolol. Another way to measure apoptotic cells is flow cytometry analysis. In this approach, detection is based on the position of PS in the cell membrane. In normal cells, most PS is localized inside of the plasma membrane, but when the cells start to undergo apoptosis, PS is relocated to the outside of the membrane and is exposed to the extracellular environment (40). The proportion of apoptotic cells can be calculated from the number of cells that bind Annexin V–FITC, which interacts with PS (40, 41). Magnolol did not change the proportion of apoptotic cells, providing further evidence that it has no influence on programmed cell death in human colon cancer cells.

Tumor metastasis is the end state of a multistep process that includes dissemination of tumor cells to distant organs, and requires cells to adapt to different tissue microenvironments (42). During metastasis, cells undergo morphological alteration called the EMT (43). Over the course of this reversible process, epithelial cells lose their polarity, preventing them from interacting with membrane surfaces, and acquire characteristics of invasive mesenchymal cells (44). The EMT is characterized by loss of cell adhesion, downregulation of epithelial markers, and upregulation of mesenchymal markers (43). We used E-cadherin, ZO-1, and claudin as representative epithelial markers. E-cadherin is a typical epithelial cell adhesion molecule, and loss of E-cadherin is thought to enable metastasis by collapsing intercellular contacts (20). ZO-1 and claudin are the main components of tight junctions, and are thus critical for the maintenance of cell polarity (45, 46). To determine the effect of magnolol on epithelial markers, we used the human colon cancer cells HCT116 and SW480. As mesenchymal markers, we examined N-cadherin, TWIST1, Slug, and Snail. N-cadherin, an adhesion molecule, is associated with invasive potential in cancers, and its overexpression promotes motility and invasion (22). TWIST1, Slug, and Snail are transcription factors that repress E-cadherin expression; thus their overexpression indicates that the EMT is already underway (47, 48). Our findings present strong evidence that magnolol effectively inhibits the EMT in human colon cancer cell lines by increasing the expression of epithelial markers and decreasing the expression of mesenchymal markers in a concentration-dependent manner. In addition, we found that magnolol significantly inhibited HCT116 cell morphological alteration and cell migration. Together, these results imply an important role for magnolol in repressing EMT and cancer progression.

Progression of colon cancer toward metastatic disease is associated with activation of the EMT, which is in turn induced by TGF-β. TGF-β signaling is mediated by ERK, GSK3β, and Smad (49, 50). As proof that magnolol disrupts TGF-β signaling, we showed that magnolol decreased expression of p-ERK, p-GSK3β, and p-Smad; in each case, the phosphorylated form of the molecule is the activated form. The EMT response to TGF-β signaling is induced by reconstitution of transcriptional activation, which promotes inactivation of genes encoding epithelial marker proteins and activation of genes encoding mesenchymal marker proteins (50). In our experiments, the TGF-β-induced EMT decreased the expression of epithelial proteins and increased the expression of mesenchymal proteins in HCT116 cells; however, magnolol restored expression of these markers to control levels. Our results strongly suggest that magnolol effectively inhibits the TGF-β-induced EMT. Moreover, TGF-β also promotes migration of individual tumor cells; consequently, inhibition of TGF-β signaling prevents the movement of single cells. Thus, magnolol blocked TGF-β signaling in HCT116 cells by inhibiting single-cell migration.

The TGF-β-induced EMT occurs at the G1/S phase of the cell cycle (27). Cell-cycle progress is driven by cyclin-dependent kinases (CDKs), which integrate mitogenic and growth signals (51). TGF-β induces cell growth arrest at G1/S by inhibiting molecules required for CDK activation, as well as by activating CDK inhibitors. When TGF-β-induced growth arrest occurs, the EMT process is initiated by attenuation of epithelial adhesion and tight junctions; subsequently, the cancer cells acquire invasive mesenchymal characteristics (26, 27, 51). Our observation that the number of cells at G1/S was lower under magnolol treatment than under treatment with TGF-β alone demonstrates that magnolol inhibited cell cycle–dependent TGF-β signaling. It means that the reason for the reduced migration of treated cells is more focused on inhibiting EMT process, which is an important physiological and morphological process in cancer cell metastasis, than on the reduction of cell growth.

The results of this study suggest a novel application of magnolol as an anti-tumor treatment in human colon cancer. One of the most important strategies in colon cancer treatment is preventing tumor metastasis, which is characterized by the EMT. Our findings provide a model for magnolol-mediated inhibition of the EMT in colon cancer. We propose that magnolol inhibits the TGF-β-induced EMT by blocking downstream TGF-β signaling and alteration of cell-cycle distribution. In addition, we confirmed that magnolol does not induce cell death in colon cancer cells, suggesting that it would have less severe side effects than apoptosis-inducing agents. Collectively, our findings provide insight into the distinct pathways associated with magnolol-mediated EMT inhibition and suggest that magnolol could be used in a rational therapeutic strategy against colon carcinoma.

## Conclusion

Colorectal cancer is a devastating disease, but therapies for this cancer remain insufficient. Moreover, the most common strategy for treating colorectal cancer involves inhibition of apoptosis, which is often associated with severe side effects. Accordingly, drugs that act via another mechanism of action are urgently required. In this paper, we demonstrate that magnolol, a natural product used in traditional Asian medicines, can prevent cancer cell invasion (an aspect of metastasis) by blocking the TGF-β-induced EMT. We suggest that magnolol inhibits the TGF-β-induced EMT by blocking downstream TGF-β signaling and alteration of cell-cycle distribution. The role of magnolol needs to be reconsidered in any future research with prevent metastasis of obvious clinical value in colorectal cancer.

## Data Availability

All datasets generated for this study are included in the manuscript/Supplementary Files.

## Author Contributions

Conceptualization: SC, H-JO, and B-YL. Methodology: SC. Software: H-JO. Validation: J-HS and Y-JS. Formal analysis: KL. Resources, supervision, project administration, and funding acquisition: B-YL. Data curation: H-JO. Writing—original draft preparation, writing—review and editing, and visualization: SC and H-JO.

### Conflict of Interest Statement

The authors declare that the research was conducted in the absence of any commercial or financial relationships that could be construed as a potential conflict of interest.
