# Supplementary figures and images for: Magnolol Suppresses TGF-β-Induced Epithelial-to-Mesenchymal Transition in Human Colorectal Cancer Cells
Source: Front Oncol. 2019 Oct 1;9:752. doi: 10.3389/fonc.2019.00752 (PMC6779771; doi:10.3389/fonc.2019.00752)

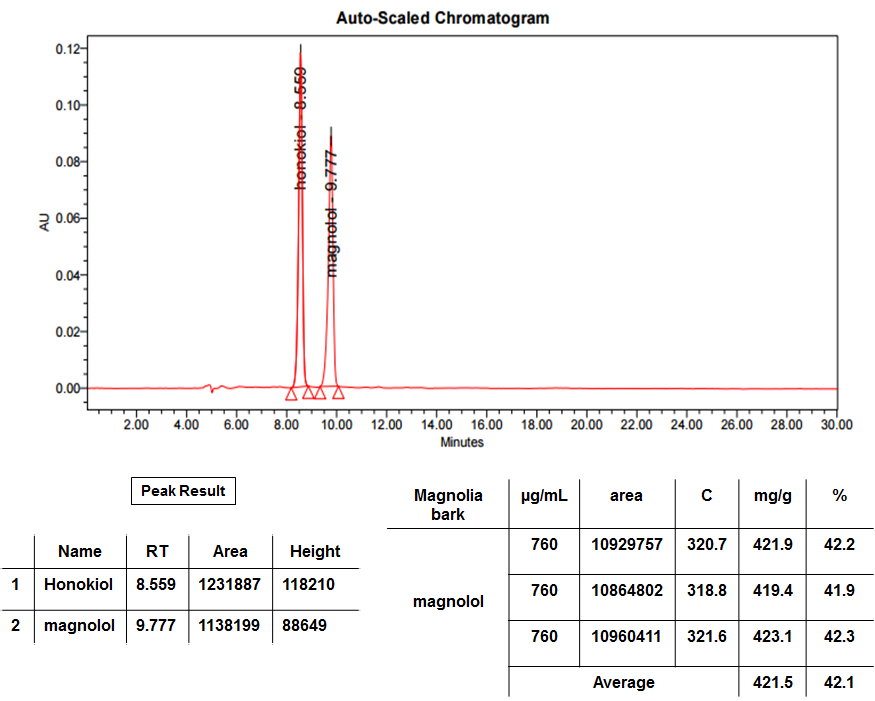

Supplement: Supplementary file 1 [file Image_1.TIF]
